# Patients at Risk for Transfusion—A Six-Year Multicentre Analysis of More Than 320,000 Helicopter Emergency Medical Service Missions

**DOI:** 10.3390/jcm12237310

**Published:** 2023-11-25

**Authors:** Christoph Jänig, Chadlia Willms, Jens Schwietring, Christoph Güsgen, Arnulf Willms, Nicole Didion, Tobias Gruebl, Dan Bieler, Willi Schmidbauer

**Affiliations:** 1 Department of Anaesthesiology and Intensive Care Medicine, Bundeswehr Central Hospital, Ruebenacher Str. 170, 56072 Koblenz, Germanytobiasgruebl@bundeswehr.org (T.G.); willischmidbauer@bundeswehr.org (W.S.); 2 Department of Medicine, ADAC Luftrettung gGmbH, Hansastr. 19, 80686 Munich, Germany; 3 Department of General, Visceral and Thoracic Surgery, Bundeswehr Central Hospital, Ruebenacher Str. 170, 56072 Koblenz, Germany; 4 Department of General, and Visceral Surgery, Bundeswehr Hospital, Lesserstr. 180, 22049 Hamburg, Germany; arnulfwillms@bundeswehr.org; 5 Department of Anaesthesiology and Intensive Care Medicine, University of Mainz Medical Centre, Langenbeckstr. 1, 55131 Mainz, Germany; didion@uni-mainz.de; 6 Department of Trauma and Reconstructive Surgery, Bundeswehr Central Hospital, Ruebenacher Str. 170, 56072 Koblenz, Germany; danbieler@bundeswehr.org

**Keywords:** emergency medical services, haemorrhagic shock, blood transfusion, massive transfusion, air ambulance

## Abstract

Background. In Europe, ambulances are increasingly being equipped with blood products for prehospital use. Available evidence on the early administration of blood products comes from military medicine and the Anglo-American medical literature; the evidence cannot be easily transferred to European countries. Objectives. This study assesses the incidence of patients with massive haemorrhage after trauma and the potential need for prehospital blood transfusions. Methods. Data reported by 37 German air rescue stations between 2015 and 2020 were retrospectively analysed to predict the need for massive transfusion. Results. A total of 320,347 helicopter emergency medical service (HEMS) missions were performed and involved 2982 patients with potential need for massive transfusion after trauma (approximately 13 transfusions per helicopter per year). Men were most affected (73%). The median age of patients was 38 years. Traffic accidents accounted for 59% of the cases. Most patients sustained multiple injuries including traumatic brain injuries (62%), as well as thoracic (54%), abdominal (39%), and extremity injuries (41%). The median “rSIG” (reversed shock index multiplied with the Glasgow Coma Scale) decreased from 4.31 to 3.78. Conclusions. Although the incidence of haemorrhagic trauma patients is low, the prehospital administration of blood products might be useful as a potentially life-saving bridging treatment until hospital admission.

## 1. Introduction

Across Europe and, increasingly, in Germany, different types of ambulances have been equipped with blood and clotting products for the early management of haemorrhagic patients [1].

The early administration of blood and clotting products is an integral part of various guidelines for the management of patients with massive haemorrhage. Treatment must address not only the need for oxygen carriers, but also early trauma-induced coagulopathy [2,3,4].

This requires the prehospital use of blood products, which, in recent, years has continuously increased. Most of the available evidence on the benefits of this approach is based on military experience. A few studies were conducted in the civilian sector as well, most of them in the United States but also in the United Kingdom [5,6,7].

There are some major differences between the study populations investigated in the literature and the situation in Europe, especially when it comes to the numbers of penetrating injuries and EMS infrastructure, which is sometimes associated with much shorter distances to receiving hospitals. Gunshot and blast injuries predominate, especially in military environments. As a result of the widespread availability and use of guns, the percentage of penetrating injuries is also considerably higher in the patient populations that were investigated in civilian studies in the United States. A comparison of EMS models of care and transport distances also reveals a number of substantial differences [6].

Consequently, the evidence on prehospital blood product administration cannot be easily transferred to the European setting. The prehospital use of blood products in trauma patient populations with a higher proportion of blunt injuries remains controversial [8,9].

The RePHILL study published in 2022 was unable to demonstrate any advantage of prehospital blood products versus normal saline [7]. The PAMPER trial reported a significantly favourable outcome for patients who received thawed plasma in the prehospital phase. In contrast, the COMBAT trial found no benefit of prehospital administered blood products in an urban environment associated with short transport times [8,9].

A systematic review from van Turenhout et al. showed no benefit from prehospital administered blood products [10]. A meta-analysis from Huang et al showed a benefit for patients in military settings. [11]

The decision to administer prehospital blood products should not be taken easily, as each transfusion also carries individual risks for the patient. It is, therefore, crucial to establish a method that enables physicians or other health care providers to identify patients with severe haemorrhage, even with limited prehospital resources.

The transfusion of a single unit of blood cannot be taken as an indicator of a relevant, life-threatening haemorrhage. However, if the patient requires a massive transfusion (10 units/24 h), it can be assumed that a relevant haemorrhage is present. It can also be assumed that these patients benefit from a prehospital transfusion if other general conditions (e.g., duration of transport, persistent uncontrolled haemorrhage) are present.

The aim of this study is to use the available mission data to retrospectively identify the annual number of patients in HEMS missions who have a high probability of requiring a massive transfusion based on a massive transfusion prediction score.

The results of this study can be used to determine whether the number of patients in the German Helicopter Emergency Medical System is high enough to justify the provision of blood products in helicopters. Furthermore, the use of the score in the prehospital phase offers an option for health care providers to identify patients with relevant haemorrhage and, if necessary, to initiate an early transfusion.

To our knowledge, there is no published data that answer the question of how many patients can be identified as being at risk for a massive transfusion already in the prehospital setting and who might benefit from an early initiation of administering blood products.

For this reason, the present study first aims to analyse prehospital data on trauma patients with a view to assess the incidence of massive haemorrhage in the HEMS setting based on HEMS missions that were conducted by different air rescue stations in all parts of Germany. Furthermore, the multicentric approach of the evaluated data considers transport times from both rural and urban areas, which better represent European conditions. The results of this study can provide a fundamental contribution to enabling a cost−benefit analysis of prehospital blood product administration for the European region.

## 2. Methods

All electronically available data that were provided by the 37 air rescue stations of the General German Automobile Club (Allgemeiner Deutscher Automobil-Club, ADAC) Air Rescue Organisation during the period from 2015 to 2020 were analysed. Missions that involved patients with any type of documented trauma were selected from all HEMS missions. Next, only patients marked as “polytrauma” were included with a view to identify patients with the most potentially severe injuries. The ADAC air rescue QM manual states that the category “polytrauma” refer to patients in whom one or the combination of several injuries resulted in a life-threatening condition.

To identify patients with a potential need for prehospital transfusion, we calculated the “rSIG-Score” (“reversed shock index”) from the first documented vital signs on scene. rSIG consists of the systolic blood pressure divided through heart rate and multiplied it with the first documented Glasgow-Coma-Scale value [rSIG = (SBP/HR) × GCS] on scene, taking hemodynamic impairment and resulting organ dysfunction into account. If the resulting product was ≤9.52, we categorized the patient as in need of transfusion, as Young TL et al. reported this as the cut-off value in their score to predict the need for massive transfusion in adult trauma patients [12].

In comparison with other known prediction scores for massive transfusions, the rSIG score can already be reliably determined preclinically. With an area under the receiving operator curve (AUROC) of 0.842 (95% CI 0.72–0.809), it also has a good predictive quality (sensitivity 0.79, specificity 0.77). Furthermore, all relevant data to calculate the score are provided in the existing electronic database, which is why we used it for this study.

**Figure 1:**
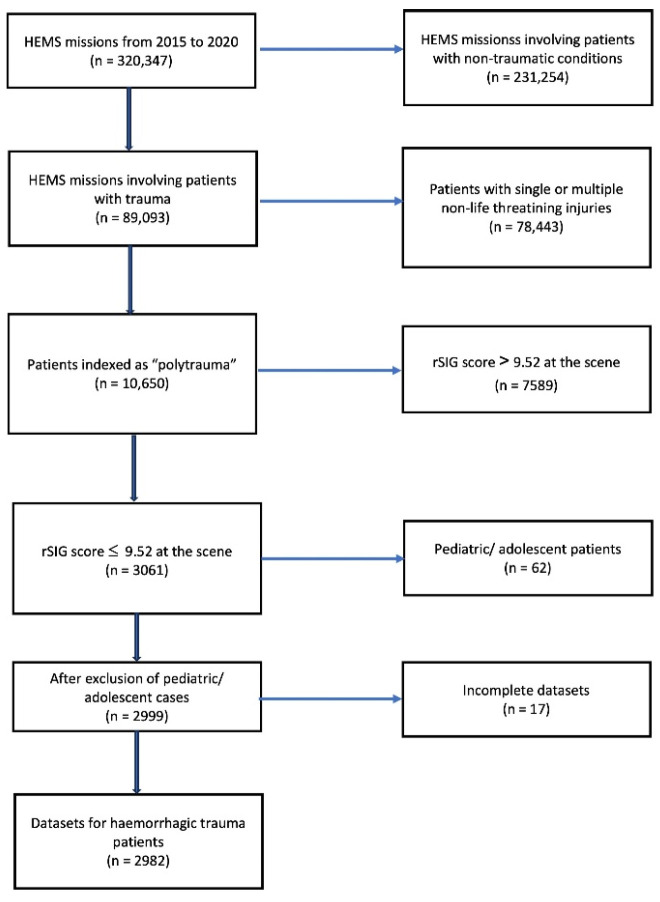
Flowchart of the identification of haemorrhagic trauma patients on the basis of data on helicopter emergency medical services (HEMS) missions from 2015 to 2020.

Children and adolescents were excluded, as the rSIG-score was only validated for adult patients. After removing incomplete datasets, a total of 2982 patients were included in the study (Figure 1).

As GCS has a decisive influence on the value of rSIG, in a further step, we excluded patients with an isolated severe traumatic brain injury, as they could falsely suggest a need for a transfusion.

The total trauma patient population and the group of haemorrhagic trauma patients were analysed using non-parametric tests (α = 0.05) and compared in terms of rSIG, systolic blood pressure, and heart rate values at the beginning and at the end of prehospital management. Descriptive statistical methods were used to analyse epidemiological data for the patient groups, injury patterns, and prehospital treatment, and transport times.

A significance level (α = 0.05) was set for the statistical analysis. A Bonferroni correction for multiple testing was applied.

The National Advisory Committee for Aeronautics (NACA) score was used as an additional instrument for the subjective assessment of injury severity.

All data that the 37 ADAC air rescue stations provided on HEMS missions for the period from 2015 to 2020 were exported from the Trace-QM platform of the ADAC Air Rescue Organization. These electronically available data correspond to the MIND 2 dataset provided in the protocol for HEMS missions. The structure of the database limits the amount of the data that can be evaluated. For example, the vital parameters are recorded at the beginning and end of patient care. However, the development of vital parameters during the course of treatment and the possible influence of therapeutic measures (e.g., effect of catecholamines) could not be derived from the available data, as they were not recorded in the electronic database.

As this is a QM database, all data were collected and stored in an anonymised format.

All data were analysed using IBM SPSS Statistics 26.0 (IBM Corp., Armonk, NY, USA).

As the study used QM data for research purposes, ethical approval was not required according to the pertinent regulations.

## 3. Results

A total of 2982 patients presented with suspected acute haemorrhage based on a rSIG score < 9.52 during this six-year period. This corresponds to an annual number of 497 patients and, in other words, to approximately 1% of a mean number of 53,324 missions per year or 4.7% of all patients classified as “polytrauma”, as indicated by the responsible HEMS physician.

Male patients were most affected (73%). The median age of patients was 38 years (IQR 17–58 years).

The leading circumstance of injury were traffic accidents (58.7%), followed by accidents at home (6.1%) and accidents at work or at school (6%). The following data were reported on the mechanisms of injury: car or truck occupant (25%), motorcyclist (17%), fall from >3 m (14%), cyclist (9%), and pedestrian struck by a vehicle (5%).

In the group of haemorrhagic trauma patients, the most common injury patterns were multiple injuries, including traumatic brain injuries (TBI) (80%) and injuries to the thorax (61%), abdomen (33%), and lower extremities (41%). Examinations by on-scene emergency physicians suggested that the pelvis was affected in 33% of the cases, the upper extremities in 34%, and the cervical spine in approximately 32%. In most cases, the injuries to the various body regions were “moderate” to “severe” (Table 1).

Based on the NACA scoring system [13], 95.1% of the haemorrhagic trauma patients had life-threatening injuries (NACA IV to V). Successful initial cardiopulmonary resuscitation during out of hospital cardiac arrest was documented in 4.1% of the patients categorized as NACA VI (n = 121). In the study group, 0.7% of the patients were categorized as NACA VII (death at the scene).

**Figure 2:**
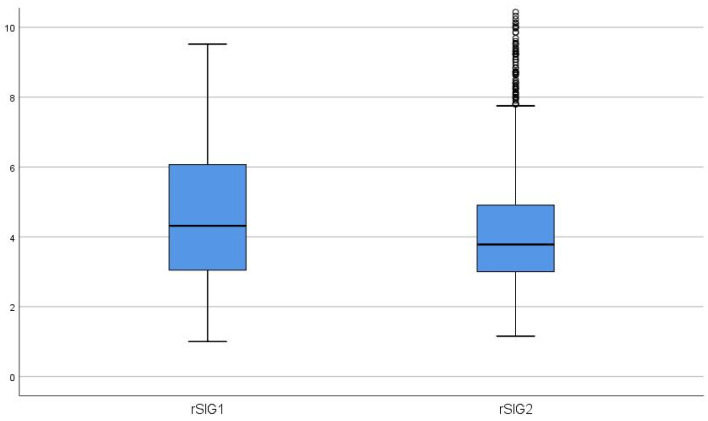
Box plots showing changes in reversed shock index × GCS value from scene [rSIG1] to arrival at the hospital [rSIG2].

The median rSIG score of 4.31 documented at arrival at the scene dropped to a median of 3.78 during hand-over at the receiving hospital. The difference between rSIG score 1 and 2 was significant (*p* < 0.001; Figure 2).

**Figure 3:**
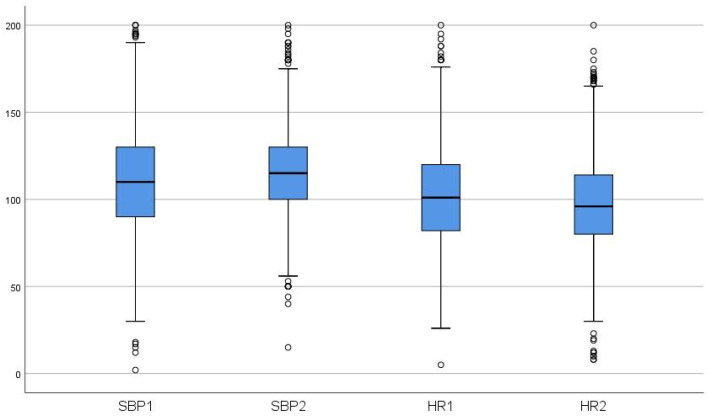
Box plots showing blood pressure and heart rate changes from the scene to arrival at the hospital. SBP 1 = systolic blood pressure at the scene; SBP2 2 = systolic blood pressure on arrival at the hospital; HR 1 = heart rate at the scene; HR 2 = heart rate on arrival at the hospital.

In the group of haemorrhagic trauma patients, the median systolic blood pressure was as low as 110 mmHg at the scene and slightly but significantly increased to 116 mmHg upon hospital admission (*p* < 0.001). The median heart rate was 100 bpm at the scene and slightly lower upon arrival at the hospital (96 bpm, *p* = 0.349) (Figure 3, Table 2).

In the prehospital phase, all patients received analgesic treatment. About 80% of the patients were intubated, in addition to a general anaesthesia. Here, 17.3% received a chest tube and catecholamines were used in 25.5% of these patients. As ultrasound was provided to all HEMS crews from 2020 and onwards, only a minority of 2.6% of the patients had a documented ultrasound examination on scene.

Following prehospital care, 81% of the patients with suspected acute haemorrhage were transported by helicopter to a hospital. In 10% of cases, patients were transferred by ground ambulance with a physician present. In 0.8% of the cases, patients were transported by ground ambulance without a physician present. Data on the mode of transport were unavailable for the remaining 8% of patients.

Patients were transported by air over a median distance of 27.5 km (13.0–44.3 km). The median prehospital care time was 48 min (95% CI 20–60 min) and included a median transport time of 18 min (95% CI 12–38 min). The median time from emergency call to admission to the resuscitation unit of the receiving hospital was 62 min (95% CI 50–71 min).

## 4. Discussion

In recent years, the administration of blood products to patients as early as possible at the scene of injury has become standard practice in the military setting and has improved primary outcomes [5]. This approach has also been increasingly used by civilian emergency medical services (EMS) in a number of countries and has been investigated in research. Many studies from the civilian sector have been published in the United States and confirmed the positive effects of the early administration of blood products on the condition of patients with massive bleeding [14,15].

There are, however, fundamental differences between Germany, on the one hand, and other European countries and the United States, on the other, when it comes to the number of trauma patients, types of injuries, and the structures and availability of medical emergency services.

The prehospital administration of blood products to patients is generally considered feasible, effective, and safe [16,17].

Although the RePHILL study was unable to show an advantage of prehospital blood product administration, the calculated study population was not reached, which, in addition to the wide CI intervals, indicates that the study was underpowered. Furthermore, composite outcomes, as used in the RePHILL trial, which combine endpoints with large variability in importance, means the results were difficult to interpret. Last, the time from randomization to hospitalization was short, potentially diluting the impact of this prehospital intervention.

Another difference between the published US military studies and the data from the RePHILL study is the use of whole blood.

Whole blood is intended to reduce the overall need for transfusion, but is not available in many European countries. No whole blood was used in the RePHILL study either. However, in the US military studies, in addition to whole blood as an initial therapy, the patients also received component therapy later on, which reduced the scientific conclusion on the effectiveness of whole blood on long-term survival [18,19].

The early use of blood and clotting products is already firmly established in a variety of guidelines. For this reason, it is no surprise that the number of European countries in which EMS ambulances carry blood products has increased as well [1].

The DGU’s annual report on trauma care in Germany from 2021 states that blood products are administered in the emergency room after an average of 50 min (1–120 min) [20]. This, in addition to the care and transport times determined in this study, could mean that the start of the transfusion can occur more than 60 min earlier. Considering the data from Sperry et al. [8], this could have a positive impact on survival after trauma. However, due to the lack of reliable data in our database, this statement cannot be conclusively supported or rejected.

There were some major differences between the study populations investigated in the literature and the situation in Europe, especially when it comes to the numbers of penetrating injuries and EMS infrastructure, which is sometimes associated with much shorter distances to receiving hospitals.

Against this background, the question arises whether it is at all possible to transfer the results of studies from the military and civilian sectors to the German system and, in other words, whether there is sufficient evidence to support the management and availability of blood and clotting products in the prehospital environment, which is a complex logistical challenge.

The objective of this study was, therefore, to estimate the incidence of massive haemorrhage in the setting of helicopter emergency medical service (HEMS) missions and to investigate relevant factors such as treatment times, injury patterns, and epidemiological data, with a view of developing a rationale for the prehospital use of blood products.

For this reason, the present study is the first to analyse data on trauma patients with a view to assess the incidence of massive haemorrhage in the HEMS setting based on many HEMS missions that were conducted by different air rescue stations in all parts of Germany.

Other parameters, such as on-scene and transport times, can help establish whether the prehospital initiation of blood product therapy provides a decisive time benefit.

The presence of haemorrhage is difficult to identify in a reliable manner on the basis of retrospective data. Scores can be useful for predicting massive transfusion requirements. Many scores have been validated based on retrospective data. Their systematic use in the present study, therefore, appears appropriate [21,22]. A limiting factor is, however, that many of the scores that are used to predict the likelihood of blood transfusion are based on parameters (e.g., specific laboratory values) that are unavailable in the prehospital setting (Table S1) [21,22,23,24,25]. In addition, ambulance equipment has changed in recent years so that ultrasound examinations can now be performed at the scene. As it was only at the end of the study period that all rescue helicopters of the ADAC Air Rescue Organization were equipped with ultrasound units, it is no surprise that ultrasound examinations were performed in a mere 2.6% of the patients investigated here. In the group of haemorrhagic trauma patients, ultrasound examinations (but not the clinical findings) were documented in 15% of the cases.

Zhu et al. reported that the combination of shock index and pulse pressure can effectively predict the need for blood transfusion [26]. As the available datasets unfortunately did not include diastolic blood pressure values, pulse pressure as an additional parameter could not be analysed, although it could be easily assessed in both the prehospital and in hospital settings and it is an independent factor associated with the need for massive transfusion [27].

In this study, injury severity was assessed based on the reversed shock index (rSI) multiplied with the GCS value, and the presence of trauma as an indicator of trauma-associated haemorrhage [12].

This methodological approach is as conservative as possible for assessing patients at risk of requiring a massive transfusion.

Furthermore, the need for massive transfusion justifies the calculated initiation of an early transfusion in the prehospital setting, as it can be assumed that the patient will benefit more from the early transfusion than suffer from the risk of an unnecessary transfusion.

Based on these criteria, the incidence of patients with a potential need for transfusions was 1%, corresponding to an annual number of approximately 497 patients. If this number of patients were distributed evenly across the number of air rescue stations considered, each HEMS team would carry out around 13 transfusions annually.

Despite the large number of EMS missions investigated here, the true incidence of haemorrhagic patients is probably higher as only 37 of a total of 86 air rescue stations in Germany were analysed, and patients who were managed by ground EMS were not included. Non-traumatic haemorrhage was also not included in this study. This assumption is supported by the fact that the proportion of patients who receive a transfusion in the emergency room is 7.8% (n = 2039). Furthermore, only 19% of the trauma patients registered in the trauma registry were admitted by helicopter [20].

On the rescue helicopter “Christoph 23” in Koblenz, which was one of the first rescue helicopters in Germany to carry prehospital blood products, 24 transfusions were carried out in 24 months. This further confirms that the retrospective analysis done in this study appeared to come close to the current operational reality.

A comparison of this incidence with other rare emergencies such as paediatric resuscitation shows that ambulances are equipped with the resources required to meet the individual prehospital care needs of patients in these similarly rare cases [28].

In the study presented here, male patients accounted for 73% of cases. This corresponds to the percentage reported in recent years from the trauma registry of the German Trauma Society (Deutsche Gesellschaft für Unfallchirurgie, DGU) [20]. This proportion has remained stable at a high level in Germany over recent years [29]. The median age of patients in this particular group was 38 years. Patients in this age group are unlikely to present with an elevated number of comorbidities, which, for example, might involve the regular use of oral anticoagulants. The mean age of patients in the group of patients investigated here, however, was considerably lower than that reported in the DGU trauma registry. Age may, thus, be a relevant outcome factor in this special group of patients with polytrauma as the analysis did not include patients who died in the prehospital setting. Included were only patients with a computable rSIG, and patients who died before arrival on the scene did not meet this criterion.

The leading circumstances of injury were traffic accidents and accidents at work.

As expected, the proportion of penetrating injuries was low (3.6%, n = 108). The body regions most commonly affected were the head (80%), thorax (61%), and abdomen (33%). In oarticular, bleeding into the major body cavities cannot be controlled in the prehospital environment. Depending on transport times, however, the early application of blood products is an effective and potentially life-saving bridging treatment until surgical bleeding control can be achieved.

Prehospital care time is defined as the time from the arrival of EMS personnel at the patient site to handover to the resuscitation team at the receiving hospital. For the group of haemorrhagic trauma patients, the median prehospital care time was 48 min and included a median transport time of 18 min. Pusateri et al. reported that prehospital plasma administration was associated with a survival benefit when transport times were longer than 20 min [30]. Shackelford et al. report that only rapid initiation of a transfusion within 15 min of the start of a military medical evacuation operation improved early survival (death within 24 h). The same study reported that the majority of deceased patients with massive haemorrhage who did not receive a prehospital transfusion died before reaching the medical treatment facility [5].

These studies suggest that an early transfusion can stabilize a patient with massive bleeding, at least long enough for the bleeding to be stopped by surgery.

At present, there is no adequate way to stop uncontrolled haemorrhage in the large body cavities in the prehospital phase. Time is of the essence to ensure patient survival. Prehospital blood products could, therefore, help to extend the time of critical bleeding, as even in a setting like the German EMS system with its high density of EMS assets, the time from injury to hospital admission is around 60 min.

If the time of the emergency call is assumed to be approximately the time of injury and the beginning of the golden hour of trauma, the data presented here show that EMS personnel require this period (a median of 62 min in this study) or even a considerably longer time to respond and manage the patient in the prehospital phase. These results also correspond to the DGU trauma registry data [28].

The data presented here provide evidence of a decrease in the rSIG score in the prehospital setting. Although this decrease can occur as a result of multifactorial reasons (e.g., reduction of SBP and GCS due to induction of anaesthesia), it may also be attributable to progressive blood loss caused by the multiple injuries sustained, as mentioned by Young TL et al. As the first two ADAC air rescue stations did not begin administering blood products (packed red blood cells and fibrinogen) until 2020, no relevant influence of the blood products on the outcome can be shown in the present study providing data from 2015–2020.

Further limitations of this study are the lack of outcome information regarding the time after hospital admission (e.g., 24 h survival, coagulopathy, and additionally received blood products) and the lack of more detailed information of treatment on site and during transport, especially with regard to the amount of fluid administered and special haemostatic procedures (e.g., REBOA and tourniquet application), as this information is not available in the database. This also means that the influence of prehospital therapy on the measured vital signs cannot be properly determined.

During the observation period, no blood products were administered prehospitally. Therefore, an effect on the outcome and on the prehospital treatment time can be observed.

Last, the provided data cannot discriminate a potentially obstructive shock—resulting from tension pneumothorax or pericardial tamponade—from hypovolemic shock, as the resulting vital signs may appear similar. However, obstructive shock is a comparatively rare pathophysiology in trauma, which can also be adequately treated prehospitally, so that it can be assumed that this does not result in a major bias.

## 5. Conclusions

In the patient population analysed here, haemorrhagic trauma patients are rare. The progressive decrease in rSIG values despite prehospital emergency treatment, however, may be indicative of a continuing need for (massive) transfusion in these patients, as the rSIG score has a good predictive quality for massive transfusion defined as 10 U of packed red blood cells/24 h. The influence of prehospital administered blood products on patient outcome is a subject of controversy. The present study is unable to contribute irrefutable evidence to this debate, as no blood products were administered during the surveyed period by ADAC HEMS crews. Assuming that the annual number of HEMS missions that involve haemorrhagic trauma patients is correctly estimated to be approximately 497 or 13 transfusions per rescue helicopter, the prehospital availability of blood products for the early treatment of haemorrhagic shock nevertheless appears necessary and justified, regarding transport times and the already proven benefit of early administration of blood products en route. However, the data provided suggest that a necessary transfusion is initiated much earlier than when first considered in the emergency department.

Because of the limitations of this study that have already been mentioned, no conclusive statement can be made about the positive influence of the prehospital use of blood products on patient survival. However, evidence of a relevant number of patients at risk for transfusion could be provided.

Further studies should address the effect of prehospital administration of blood on patient condition (e.g., vital signs, lactate, and base excess) upon admission to the hospital as short-term outcome and regarding length of stay, overall morbidity, and survival to discharge as long-term outcomes.

## Figures and Tables

**Table 1:** Patterns and severity of injuries to different body regions in the group of haemorrhagic trauma patients.

	Traumatic Brain Injury	Cervical Spine	Thorax	Abdomen	Pelvis	Upper Extremity	Lower Extremity
Total	80%	32%	61%	33%	33%	34%	41%
Minor injury	3.3%	2.2%	1.8%	1.6%	1.6%	3.4%%	2.8%
Moderate injury	17.7%	17%	25.8%	8%	15%	20.5%	17.2%
Severe injury	58.6%	11.3%	33.8%	16.4%	17.4%	10.3%	21.1%

**Table 2:** Changes in reversed shock index × GCS (rSIG), systolic blood pressure (SBP), and heart rate (HR) from the scene to arrival at the hospital SBP 1 = systolic blood pressure at the scene; SBP 2 = systolic blood pressure on arrival at the hospital; HR 1 = heart rate at the scene; HR 2 = heart rate on arrival at the hospital.

	At the Scene			Arrival at Hospital		
	rSIG 1	HR 1	SBP 1	rSIG 2	HR 2	SBP 2
		[bpm]	[mmHg]		[bpm]	[mmHg]
Median	4.31	100	110	3.78	96	116
25th percentile (Q1)	3.04	82	90	3.0	80	100
75th percentile (Q3)	6.06	120	130	4.90	113	130

## Data Availability

The data that support the findings of this study are available from ADAC Luftrettung, but restrictions apply to the availability of these data, which were used under license for the current study, and are thus not publicly available. Data are, however, available from the authors upon reasonable request and with permission from ADAC Luftrettung.

## Supplementary Materials

The following supporting information can be downloaded at: https://www.mdpi.com/article/10.3390/jcm12237310/s1, Table S1: Overview of scores for assessing individual transfusion needs.

## Abbreviations

ABC	Assessment of Blood Consumption
ADAC	Allgemeiner Deutscher Automobilclub; General German Automobile Club
DGU	Deutsche Gesellschaft für Unfallchirurgie German Trauma Society
EMS	Emergency Medical Service
ETS	Emergency Transfusion Score
FAST	Focused Assessment Sonography for Trauma
Hb (g/dL)	Haemoglobin
HEMS	Helicopter Emergency Medical Service
HR	Heart rate
MASH	Military Acute Severe Haemorrhage
MOI	Mechanism of injury
mTICCS	Modified Trauma-Induced Coagulopathy Clinical Score
NACA	National Advisory Committee for Aeronautics
PWH	Prince of Wales Hospital
RABT	Revised Assessment of Bleeding and Transfusion
rSIG RR	Reversed shock Index × GCS Respiratory rate
SBP	Systolic blood pressure
SI	Shock index
TASH	Trauma-Associated Severe Haemorrhage
