# Supplementary material for: Patients at Risk for Transfusion—A Six-Year Multicentre Analysis of More Than 320,000 Helicopter Emergency Medical Service Missions
Source: J Clin Med. 2023 Nov 25;12(23):7310. doi: 10.3390/jcm12237310 (PMC10706994; doi:10.3390/jcm12237310)
Supplement: Supplementary file 1 [file jcm-12-07310-s001.zip › jcm-2690452-supplementary.pdf]

Supplementary Table S1. Overview of scores for assessing individual transfusion needs.

	TASH		PWH		Vandromme		ABC		Schreiber		Larson		MASH		mTICCS		RABT		ETS	
	**Value**	**Points**	**Value**	**Points**	**Value**	**Points**	**Value**	**Points**	**Value**	**Points**	**Value**	**Points**	**Value**	**Points**	**Value**	**Points**	**Value**	**Points**	**Value**	**Points**
Age																			20–60	0.5
																			>60	1.5
Gender	male	1																		
Head/ neck															yes	1				
upper extrimity															yes	1				
lower extrimity															yes	1				
No amputated limbs													number	1–4						
Haemothorax													yes	1						
Torso															yes	2				
Abdomen															yes	2				
Pelvic fracture	unstable	6	displaced	1									severe inj.	1	yes	2	yes	1	unstable	2
femoral fracture	open/ displaced	3	O TC +	2									number	1–2						
open tibial fracture													number	1–2						
sever arterial bleeding													yes	1						
FAST pos.	pos	3					pos.	1					pos	1			pos.	1	pos.	2
Respiratory Rate													≥30	1						
HR (bpm)	≥120	2	≥120	1	>105	1	≥120	1			>110	1	≥100	1						
SBP	100–119	1			<110	1					<110	1	<110	1					90–120	1.5
	<100	4																	<90	2.5
			≤90	1			≤90	1							yes	5				
SI																	>1	1		
Hb (g/dl)	<12	2	7.1–10	1	<11	1			<11	1	<11	1								
	<11	3	≤7	10																
	<10	4																		
	<9	6																		
	<7	8																		
Base Excess (mmol/l)	<−2	1	>5	1							≤−6	1								
	<−6	3																		
	<−10	4																		
MOI							penetrating	1	penetrating	1							penetrating	1	traffic accident	1
																			fall > 3 m	1
Resus Room + TTA															yes	2				
Admission from scene																			yes	1
INR					> 1.5	1			>1.5	1										
GCS			8	1									<15	1						
Lactate					≥ 5	1														

PH treatment	PH BloodPr	1
	TQ	1
	PH/ ED haemost. Agent	1
